# Similar Genetic Architecture with Shared and Unique Quantitative Trait Loci for Bacterial Cold Water Disease Resistance in Two Rainbow Trout Breeding Populations

**DOI:** 10.3389/fgene.2017.00156

**Published:** 2017-10-23

**Authors:** Roger L. Vallejo, Sixin Liu, Guangtu Gao, Breno O. Fragomeni, Alvaro G. Hernandez, Timothy D. Leeds, James E. Parsons, Kyle E. Martin, Jason P. Evenhuis, Timothy J. Welch, Gregory D. Wiens, Yniv Palti

**Affiliations:** ^1^National Center for Cool and Cold Water Aquaculture, United States Department of Agriculture, Agricultural Research Service, Kearneysville, WV, United States; ^2^Animal and Dairy Science Department, University of Georgia, Athens, GA, United States; ^3^High-Throughput Sequencing and Genotyping Unit, Roy J. Carver Biotechnology Center, University of Illinois at Urbana-Champaign, Urbana, IL, United States; ^4^Troutlodge, Inc., Sumner, WA, United States

**Keywords:** aquaculture, bacterial cold water disease, genome-wide association study, quantitative trait loci, rainbow trout

## Abstract

Bacterial cold water disease (BCWD) causes significant mortality and economic losses in salmonid aquaculture. In previous studies, we identified moderate-large effect quantitative trait loci (QTL) for BCWD resistance in rainbow trout (*Oncorhynchus mykiss*). However, the recent availability of a 57 K SNP array and a reference genome assembly have enabled us to conduct genome-wide association studies (GWAS) that overcome several experimental limitations from our previous work. In the current study, we conducted GWAS for BCWD resistance in two rainbow trout breeding populations using two genotyping platforms, the 57 K Affymetrix SNP array and restriction-associated DNA (RAD) sequencing. Overall, we identified 14 moderate-large effect QTL that explained up to 60.8% of the genetic variance in one of the two populations and 27.7% in the other. Four of these QTL were found in both populations explaining a substantial proportion of the variance, although major differences were also detected between the two populations. Our results confirm that BCWD resistance is controlled by the oligogenic inheritance of few moderate-large effect loci and a large-unknown number of loci each having a small effect on BCWD resistance. We detected differences in QTL number and genome location between two GWAS models (weighted single-step GBLUP and Bayes B), which highlights the utility of using different models to uncover QTL. The RAD-SNPs detected a greater number of QTL than the 57 K SNP array in one population, suggesting that the RAD-SNPs may uncover polymorphisms that are more unique and informative for the specific population in which they were discovered.

## Introduction

Bacterial cold water disease (BCWD) inflicts substantial mortality and economic losses in salmonid fish aquaculture (Nematollahi et al., 2003; Barnes and Brown, 2011). The etiological agent of BCWD is a gram-negative bacterium, *Flavobacterium psychrophilum (Fp)*, and existing methods to control BCWD outbreaks are inadequate. At the National Center for Cool and Cold Water Aquaculture (NCCCWA), we have developed a selective breeding program to improve the genetic resistance of rainbow trout to BCWD and have shown that BCWD resistance has a moderate heritability and responds to selection (Leeds et al., 2010). Furthermore, we revealed the complex genetic architecture of BCWD resistance (Vallejo et al., 2010) and identified several moderate-large effect quantitative trait loci (QTL) for this trait in the NCCCWA odd- and even-year rainbow trout selective-breeding populations (Wiens et al., 2013; Vallejo et al., 2014a; Liu et al., 2015b; Palti et al., 2015b). While those QTL can be fine mapped to identify positional candidate genes, the complex genetic architecture of BCWD resistance and high genetic variability we discovered in past studies (Vallejo et al., 2014a) suggest that whole genome-enabled selection is more effective for improving genetic resistance against BCWD in rainbow trout aquaculture, and we were able to empirically demonstrate that whole genome-enabled selection can double the accuracy of predicted genetic merit of potential breeders compared to traditional family-based selection (Vallejo et al., 2017).

For agricultural livestock species, single nucleotide polymorphism (SNP) chips have been the platform of choice for whole genome genotyping of at least 50 K SNPs (Matukumalli et al., 2009; Ramos et al., 2009; Groenen et al., 2011); as well as the recently developed 57 K SNP chip for rainbow trout (Palti et al., 2015a). However, sequencing-by-genotyping methods that do not require *a priori* marker discovery or a reference genome sequence and are capable of concurrent marker discovery and genotyping in many individuals were developed for genetic analyses (Davey et al., 2011). One such technique is restriction-site-associated DNA (RAD) sequencing (Miller et al., 2007; Baird et al., 2008). In recent years, the method of RAD genotyping by sequencing has been widely used in salmonid species for SNP discovery, generating linkage maps, QTL mapping, genome-wide association studies (GWAS) and for evaluating genome-enabled selection (Hecht et al., 2012, 2013; Houston et al., 2012, 2014; Miller et al., 2012; Hale et al., 2013; Narum et al., 2013; Brieuc et al., 2014; Campbell et al., 2014; Gonen et al., 2014; Palti et al., 2014, 2015b; Liu et al., 2015a,b; Vallejo et al., 2016).

A number of moderate-large effect QTL associated with BCWD resistance have been found on 24 of the 29 rainbow trout chromosomes using linkage analysis mapping (Johnson et al., 2008; Wiens et al., 2013; Palti et al., 2014; Quillet et al., 2014; Vallejo et al., 2014a) and GWAS methods (Campbell et al., 2014; Liu et al., 2015b; Palti et al., 2015b). However, these previous studies had limitations on QTL detection. First, most of the described genome-wide association analyses have tested one-SNP at a time using single-regression or mixed linear models with a fixed SNP effect along with a random polygenic effect to capture the effects of all other genes. While those studies have been successful in detecting associations, those associations have typically explained only a small fraction of the trait genetic variance (Visscher et al., 2010). Conversely, in GWAS using whole-genome selection models that simultaneously fit all markers as random effects, the markers jointly explain a larger fraction of the genetic variance which highlights the utility of using multiple-regression GWAS models with the SNPs joined into genomic windows for accurate QTL mapping (Hayes et al., 2010; Fan et al., 2011; Onteru et al., 2011). Second, most of the reported QTL for BCWD resistance were identified using linkage-based methods and GWAS was performed within individual segregating families with relatively small sample size and low detection power. Consequently about half of the reported findings from such QTL mapping studies are expected to represent false positives (Higginson and Munafo, 2016). Third, the previous studies used lower density genotyping platforms and did not have a reference genome sequence (GenBank Assembly Accession GCA_002163495) for accurate prediction of the order and physical proximity of the genetic markers. Furthermore, to our knowledge, in the current study we are using for the first time SNP genotype data with the genome physical map coordinates for GWAS in rainbow trout.

There is ambiguity on the best computational algorithm when using multiple-regression based models in genomic selection (GS) and GWAS experiments. The genetic architecture of the trait and the population structure can have a major impact on the accuracy of the genomic predictions and estimated marker effects. Therefore, it is essential to compare the performance of the best competing algorithms on GS and GWAS when studying a complex inheritance trait for the first time in a population. This will allow effective discovery of QTL underlying the genetic basis of the complex trait and control the type I error rate, which is often high in genome-wide discovery experiments.

In multiple-regression based GWAS models that fit all the markers altogether, the genomic BLUP (GBLUP) method assumes that the trait genetic variance is controlled by the polygenic inheritance of an infinite number of minute effect loci and thus uses all the markers data to calculate the genomic relationship (G) matrix. Conversely, the Bayesian variable selection model assumes that the trait genetic variance is explained by a relatively low number of loci each with small-moderate or large effect (Habier et al., 2007; Hayes et al., 2009; de Los Campos et al., 2013; Fernando and Garrick, 2013; Howard et al., 2015). Based on the underlying assumptions of these models, the GBLUP model is expected not to perform as well as the Bayesian variable selection model when the trait is controlled by few moderate-to-large effect QTL. The GBLUP method was extended into two methods: (i) single-step GBLUP (ssGBLUP) method which combines the pedigree-based (A) and genomic relationship (G) matrices into the H relationship matrix (Aguilar et al., 2010; Legarra et al., 2014); and (ii) weighted single-step GBLUP (wssGBLUP) method which mimics the Bayesian variable selection model by fitting in the multiple-regression model only SNPs that explain moderate-large fraction of the trait genetic variance (Wang et al., 2012).

The recent development of the 57 K SNP array (Palti et al., 2015a), a dense genetic linkage map with 47,939 SNP markers (Gonzalez-Pena et al., 2016), and the release of the improved rainbow trout reference genome (GenBank Assembly Accession GCA_002163495) have provided the needed tools for performing GWAS to identify genomic regions associated with BCWD resistance in rainbow trout. The main objectives of this study were to (1) identify and validate QTL associated with BCWD resistance in two commercially-relevant rainbow trout breeding populations; (2) characterize the genetic architecture of rainbow trout resistance to BCWD; (3) compare the QTL mapping efficiency and determine whether the Chip-SNP and RAD-SNP genotyping platforms detect the same QTL; and (4) compare the QTL mapping efficiency of two widely-used multiple-regression GWAS models.

## Materials and methods

### Rainbow trout rearing and BCWD challenge

Protocols for this study were reviewed and approved by the NCCCWA Institutional Animal Care and Use Committee (Kearneysville, WV).

Details of the 21-day BCWD challenge have been reported elsewhere (Silverstein et al., 2009; Leeds et al., 2010). Briefly, “forty fish from each family were moved into 2.4-L challenge tanks (1 family/tank) in an isolated challenge facility at ~75 days post-hatch. Two replicate challenge tanks were used for each family. After moving to the challenge tanks, fish were given a 1-wk acclimation period and they were fed once daily to apparent satiation during the acclimation period. Challenge tanks were supplied with 1.9 L/min of flow-through spring water, and water temperature was ~12.9°C. Previously, a bank of frozen aliquots was prepared from the culture of a single bacterial clone from a virulent strain of *F. psychrophilum* (CSF259-93) and stored at −80°C as 10% glycerol stock. An aliquot was cultured on tryptone yeast extract with salts agar plates for 5 days at 15°C. Bacterial cells were harvested, re-suspended in PBS, and diluted to an optical density of 0.250 measured at 525 nm. Serial dilutions of the bacterial suspension were re-cultured on tryptone yeast extract with salts agar plates on the same day as the challenge to determine actual cfu per inoculation. Fish were anesthetized in 100 mg/L of tricaine methanesulfonate (Tricaine-S, Western Chemical, Ferndale, WA) and injected intraperitoneally with 100 μL of the bacterial suspension. The post-hatch age of fish at the time of challenge was ~84 d” (Leeds et al., 2010). Mortalities were removed and recorded daily and fin clipped. Fish that survived the challenge were euthanized in a lethal dose of Tricaine methane sulfonate (Tricaine-S, Western Chemical, Inc., Ferndale, WA) and fin clipped. Fin clips from all fish (mortalities and survivors) were individually kept in 95% ethanol until DNA was extracted using established protocols (Palti et al., 2006).

### Rainbow trout populations used in GWAS

Fish used in this study were sampled from two populations, and all analyses were performed separately by population. The first population included fish with genotypes and phenotypes from 10 full-sib (FS) families randomly sampled from a total of 71 pedigreed FS families with phenotype data from year-class (YC) 2005 of the NCCCWA BCWD resistant line (NCCCWA) and it was described in our previous GS study (Vallejo et al., 2016). Briefly, the YC 2005 families represented the base generation of the breeding line, and thus had not previously been selected for BCWD resistance. Each family had *n* = 39–80 fish evaluated in the laboratory BCWD challenge in one or two tanks per family. The phenotypic dataset included disease resistance phenotypes from *n* = 4,492 fish from 71 FS families (Table 1), and the pedigree file included 4,659 records. In this NCCWA sample, a total of *n* = 583 fish had both genotype and phenotype records. Following pedigree quality control, the original NCCCWA sample of *n* = 583 genotyped fish was reduced into *n* = 577 because four fish were flagged as duplicated or cloned samples by the QC pipeline and two fish did not assign to the expected family based on the pedigree records.

**Table 1:** Experimental variables of GWAS conducted in two rainbow trout populations using two SNP genotyping methods.

**Population^a^**	**Genotyping platform^b^**	**GWAS method^c^**	**BCWD phenotype^d^**	**1 Mb windows**	**Genotyped SNP^e^**	**Genotyped fish^e^**	**Phenotyped fish**	**Genetic parameter^f^**			
								$\sigma_{g\;}^{2}$	$\sigma_{c}^{2}$	$\sigma_{e}^{2}$	$h_{M}^{2}$
TLUM	Chip	BayesB	DAYS	1,840	31,787	1,473	1,473	13.72	Na^g^	44.56	0.24
TLUM	Chip	BayesB	STATUS	1,840	31,787	1,473	1,473	0.58	Na	1.00	0.23
TLUM	Chip	wssGBLUP	DAYS	1,394	31,787	2,500	7,893	15.55	0.50	32.48	0.32
TLUM	Chip	wssGBLUP	STATUS	1,406	31,787	2,500	7,893	1.24	0.03	1.00	0.35
NCCCWA	Chip	BayesB	DAYS	1,847	36,666	577	577	13.30	Na	35.71	0.27
NCCCWA	Chip	BayesB	STATUS	1,847	36,666	577	577	0.79	Na	1.00	0.28
NCCCWA	Chip	wssGBLUP	DAYS	1,420	36,666	652	4,492	13.09	0.23	30.95	0.30
NCCCWA	Chip	wssGBLUP	STATUS	1,408	36,666	652	4,492	0.83	0.01	1.00	0.28
NCCCWA	RAD	BayesB	DAYS	1,777	7,972	574	574	13.40	Na	34.88	0.28
NCCCWA	RAD	BayesB	STATUS	1,777	7,972	574	574	0.86	Na	1.00	0.29
NCCCWA	RAD	wssGBLUP	DAYS	1,243	7,972	649	4,492	15.09	0.20	29.71	0.34
NCCCWA	RAD	wssGBLUP	STATUS	1,253	7,972	649	4,492	1.06	0.01	1.00	0.32

a *GWAS was performed using fish from Troutlodge US May (TLUM) and NCCCWA rainbow trout populations, separately*.

b *The sampled fish were genotyped with the 57 K SNP array (Chip) and with RAD-SNPs (RAD) generated by sequencing of RAD tag libraries*.

c *GWAS was performed using Bayesian variable selection model BayesB and weighted single-step GBLUP at iteration 2 (wssGBLUP) methods. The BayesB method used 1 Mb exclusive-consecutive windows and the wssGBLUP method used 1 Mb moving-sliding windows*.

d *BCWD resistance phenotypes: survival days after disease challenge (DAYS) and binary fish survival status (STATUS)*.

e *These are effective number of genotyped SNPs and fish after data quality control, respectively, used in the GWAS analyses*.

f *Genetic parameter estimates: $\sigma_{g\;}^{\text{2}}$is the additive genetic variance; $\sigma_{c}^{\text{2}}$ is the common environment variance; $\sigma_{e}^{\text{2}}$ is the residual error; and $h_{M}^{\text{2}}$ is the proportion of phenotypic variance explained by the markers. For the binary phenotype STATUS, the $h_{M}^{\text{2}}$ estimated on the underlying scale of liability was transformed to the observed scale*.

g *Na indicates a non-available estimate. The BayesB GWAS model did not include the common environment random effect*.

The second population included 102 pedigreed FS families from YC 2013 of the commercial breeding company Troutlodge, Inc., May-spawning population (TLUM) and it was described in our previous GS study (Vallejo et al., 2017). Briefly, the original study design was to sample *n* = 1,500 fish with phenotypes and genotypes from 50 FS families; in practice, a total of *n* = 1,473 fish had both phenotype and genotype records from those 50 FS families (*n* = 17–40 fish per family). The 102 YC 2013 families represented a commercial nucleus breeding population undergoing selection for growth, and thus had not previously been selected for BCWD resistance. The fish were evaluated for BCWD survival in the laboratory challenge in two tanks per family with an initial stocking of 40 fish per tank. The phenotypic dataset included BCWD disease phenotype records from *n* = 7,893 fish from 102 FS families (Table 1), and the pedigree file included 32,279 records. A summary of the experimental variables of GWAS conducted with fish sampled from these two rainbow trout populations is presented in Table 1.

### BCWD resistance phenotypes

The discrete BCWD resistance phenotype DAYS, the number of days post-challenge until the fish succumbed to the disease, was recorded for all mortalities and survivors were assigned a value of 21. Each fish also had a binary survival STATUS record. The BCWD resistance phenotype STATUS had two categories: 1 = the fish died during the 21 days post-challenge evaluation period; and 2 = the fish survived for the duration of the challenge. The DAYS and STATUS records were analyzed separately using univariate GWAS models described below. We tested these traits for normal distribution even though knowing a priori that these are disease survival traits that are expected not to have a perfect fit to a normal distribution. As expected, generally, we find that the survival DAYS is closer to a normal distribution, but still short of being considered a normally distributed trait. The binary disease STATUS does not have a normal distribution.

### SNP genotyping platforms

The fish sampled from TLUM and NCCCWA populations were genotyped using the Rainbow Trout Affymetrix 57 K SNP array (Chip) following previously described procedures (Palti et al., 2015a) and the samples were genotyped by a commercial service provider (Geneseek, Inc., Lincoln, NE) following the Axiom genotyping procedures described by the array manufacturer (Affymetrix). The quality control (QC) bioinformatics pipeline applied to the Chip-SNP genotype data collected in the TLUM (Vallejo et al., 2017) and NCCCWA (Vallejo et al., 2016) populations were already described. After genotype data QC, a total of 41,868 and 49,468 SNPs were included in the TLUM and NCCCWA raw Chip genotype datasets, respectively.

The fish sampled from the NCCCWA population were also genotyped by sequencing of restriction-site-associated DNA (RAD) tag libraries as we have previously described (Vallejo et al., 2016). After genotype data QC, a total of 24,465 RAD-SNPs were included in the raw RAD genotype dataset. The raw sequence data from the RAD libraries were deposited in the NCBI SRA database (Accession SRP063932). The sequences of the RAD loci, SNP alleles and the SNP position in the genome are provided in Table S1.

Before performing GWAS analyses, the raw marker genotype datasets were further QC filtered using algorithms implemented in the software BLUPF90 (Misztal et al., 2015). For the Chip data, the QC retained SNPs with a genotype calling rate higher than 0.90, minor allele frequency higher than 0.05, and departures from Hardy-Weinberg equilibrium less than 0.15 (difference between expected and observed frequency of heterozygotes). Parent-progeny pairs were tested for discrepant homozygous SNPs, and SNPs with a conflict rate of more than 1% were discarded from further analysis. For the RAD data, the QC retained SNPs with a genotype calling rate higher than 0.70. Following this final QC step, 33,838 SNPs, 39,112 SNPs, and 9,534 SNPs were retained for analyses of the TLUM, NCCCWA (Chip) and NCCCWA (RAD) datasets, respectively. Next, we determined the physical map position (GenBank Assembly Accession GCA_002163495) of each of the QC filtered markers and found that a small fraction did not have a physical map location. The numbers of effective genotyped markers and effective genotyped fish that were used with each specific GWAS model and genotyping platform in the evaluated populations are shown in Table 1.

### GWAS with bayesian variable selection model BayesB

This study was conducted to identify chromosomal regions that have the greatest impact on the variation of BCWD resistance because it is difficult to infer individual SNP effects from a multiple regression model that fits markers simultaneously at a genome-wide scale (Garrick and Fernando, 2013). Therefore, instead of using the markers effect to make an inference on a particular locus, we used the markers effect to make an inference about a particular genomic region that encompasses a number of contiguous loci in association with the trait (Fan et al., 2011; Fernando et al., 2014). Thus, the GWAS analysis was performed separately for each population and BCWD resistance phenotype using multiple-regression GWAS models that use simultaneously all the SNPs in the association test.

In the GWAS analysis, we used the Bayesian variable selection model BayesB (Fernando and Garrick, 2009). Before GWAS searching for genomic regions associated with BCWD resistance, we tested 0.5 and 1 Mb exclusive-windows in GWAS with BayesB model and found that 1 Mb windows provided Manhattan plots with less noisy baseline which was in agreement with other GWAS reports (Kizilkaya et al., 2013; Saatchi et al., 2013); so we decided to use 1 Mb exclusive-windows in the GWAS performed with BayesB model. This model uses only fish that had both genotype and phenotype records. The TLUM population sample included *n* = 1,473 fish from 50 YC 2013 families with phenotype and genotype records, and the NCCWA sample included *n* = 577 fish from 10 YC 2005 families with phenotype and genotype records (Table 1).

The GWAS for DAYS used this linear model: *y* = 1μ+*Zα*+*e*; where *y* is *n x 1* vector of phenotypic records; 1 is a vector of all ones; μ is overall mean of phenotypic records; *Z* is an *n x k* matrix of genotype covariates (coded as −10, 0, or 10) for *k* SNP markers, α is a *k x 1* vector of random regression coefficients of *k* additive marker effects, and *e* is a vector of residuals. The implementation of GENSEL internally divides by 10 the marker effects so there is nil impact on the variance estimates (Dorian Garrick, personal communication). The genotype and phenotype records were used to estimate markers effect using the Bayesian variable selection model BayesB implemented in the software GENSEL (Fernando and Garrick, 2009) as we have previously described (Vallejo et al., 2016). The GWAS for the binary phenotype STATUS was conducted using the categorical analysis routine implemented also in GENSEL software (Fernando and Garrick, 2009, 2013; Garrick and Fernando, 2013).

The two population datasets have been published already in two GS studies (Vallejo et al., 2016, 2017) in which we explain our decision not to use TANK/FAMILY as fixed effect in the GS models run with BayesB. Briefly, in the analyses of these datasets with BayesB, we have tested the idea of including “tank/family” as fixed effect in the model to pre-correct the response variables in the study. In doing so, we have consistently found that the accuracy of genetic predictions will be reduced dramatically when including “tank/family” as fixed effect in the model with BayesB. This happens due to limitations imposed by the design of our disease challenge studies: in our experiments with young/small fish, the tank and family effects are confounded due to rearing of one family progeny fish per tank; thus, even though each family was replicated in two tanks, when including “tank/family” as a fixed effect in the model it takes away needed genetic variance for the GS analysis.

The BayesB model fits a mixture model to estimate marker effects assuming there are two types of SNP markers: a fraction of non-zero SNP effects (1−π) that are drawn from distributions with marker-specific variance $\left(\sigma_{\alpha }^{2}\right)$, and other known fraction of markers (π) that have zero effect on the quantitative trait (Meuwissen et al., 2001). In this study, the mixture parameter π was assumed to be known and defined to meet the condition *k* ≤ *n*; where *n* is the number of fish with genotype records, *p* is the effective number of SNPs, and *k* = (1 −π) *p* is the number of markers sampled as having non-zero effect that are fitted simultaneously in the Bayesian multiple regression model (Garrick and Fernando, 2013). For each of the evaluated datasets, we tested empirically three π parameter values that meet the condition *k* ≤ *n* and identified the mixture parameter π that estimated GEBVs with the highest accuracy using progeny performance data with procedures shown elsewhere (Vallejo et al., 2016, 2017). We then decided to use π values of 0.97 and 0.99 with TLUM-Chip and NCCCWA samples, respectively, in the GWAS analysis with BayesB because these π values yielded best accuracy genomic predictions (Results not presented).

The BayesB model uses Gibbs sampling method in the GWAS analysis (Garrick and Fernando, 2013). In this study, the BCWD phenotype records were analyzed using 270,000 Markov Chain Monte Carlo (MCMC) iterations from which the first 20,000 samples were discarded as burn-in; from the remaining 250,000 samples, we saved one from every 50 samples so the marker effects and variances were estimated as the posterior means of collected 5,000 independent samples. The proper mixing and convergence of the MCMC iterations were assessed with the R package CODA (Plummer et al., 2006).

The window variances can be viewed as post-processing (Fernando and Garrick, 2013; pp. 258–272). The “vare” and “varEffects” are calculated. The “vare” is based on all the residuals and the residuals are based on all the effects in the model at this iteration of the chain. The “varEffects” is based on all the alphas or marker effects (BayesC), regardless of “windows,” or is unique for each locus in BayesA and BayesB (“vare” in R code).

### GWAS with weighted single-step GBLUP model

We performed GWAS with the weighted single-step GBLUP (wssGBLUP) method (Wang et al., 2012; Misztal et al., 2015). We also used 1 Mb inclusive or sliding-windows in GWAS performed with wssGBLUP which we found are more informative than exclusive-windows (Results not presented). The GWAS analysis with wssGBLUP, in contrast to BayesB, uses all available information on sampled fish such as pedigree, genotype and phenotype records including those fish that had only phenotypic records (i.e., those with missing genotype data) as long as the sampled fish are pedigree related (Aguilar et al., 2010; Christensen and Lund, 2010). The TLUM sample included *n* = 7,893 fish from 102 YC 2013 families with phenotype records, and the NCCWA sample included *n* = 4,492 BCWD fish from 71 YC 2005 families with phenotype records (Table 1).

In GWAS with wssGBLUP, the weights for each SNP are 1's for the 1st iteration which means that all SNPs have the same weight (i.e., standard single-step GBLUP). For the next iterations (2nd, 3rd, etc.), the weights are SNP specific variances that are estimated using both the SNP allele-substitution effect from the previous iteration and their corresponding allele frequencies (Wang et al., 2012). In this study, we decided to use results from the 2nd iteration because they provide the highest accuracy genomic predictions (Vallejo et al., 2016) and marker effects (Wang et al., 2012; Irano et al., 2016; Melo et al., 2016).

In GWAS with TLUM population sample, we fitted linear and threshold models for DAYS and STATUS, respectively. We used the following animal model: *y* = 1μ+*Za*+*Wc*+*e*; where 1 is a vector of all ones; μ is overall mean of phenotypic records; *a* is a vector of random individual animal effects, *c* is a vector of random common environment effects and *e* is a vector of residual effects; and *Z* and *W* are incidence matrices relating records to random animal and common environment effects in *a* and *c*, respectively. The variances of *a, c* and *e* are:

(1) $$var\left[\begin{array}{c} a\\ c\\ e \end{array}\right]=\left[\begin{array}{ccc} H\sigma_{a}^{2} & 0 & 0\\ 0 & I\sigma_{c}^{2} & 0\\ 0 & 0 & I\sigma_{e}^{2} \end{array}\right];$$

where $\sigma_{a}^{2}$, $\sigma_{c}^{2}$ and $\sigma_{e}^{2}$ are total genetic additive, common environment and residual variances, respectively, and *H* is a matrix that combines pedigree (*A*) and genomic (*G*) relationship matrices as in Aguilar et al. (2010), and its inverse as defined in Wang et al. (2012). The full-sib fish progeny from each family was allocated into two tanks for BCWD challenge evaluation, so the variable tank nested within family was used to model the common environment effect. The linear and threshold models used with the NCCCWA sample were similar to those used with the TLUM sample. The linear model for survival DAYS and threshold model for the binary survival STATUS were fitted using computer applications implemented in the software BLUPF90 (Misztal et al., 2015). The binary disease STATUS was analyzed with a threshold model using a Bayesian method that had a single chain with a total of 270,000 iterations; the first 20,000 iterations were discarded as burn-in iterations; then from the remaining 250,000 samples, one from every 50 samples were saved. Thus, 5,000 independent samples were used in the GWAS analysis. The correct mixing and convergence of these MCMC iterations were diagnosed using the R package CODA (Plummer et al., 2006).

We decided not to use sophisticated survival analysis models in the analysis of the binary STATUS, because our group and others have shown that the genetic parameters, family EBVs, accuracy of selection and accuracy estimates of each solution were not different between the survival and linear animal models (Leeds et al., 2010). In this study, we found empirically that the genomic predictions for the binary STATUS are more accurate with a threshold model than with a simple linear model; thus, we decided to use a threshold model with the binary disease STATUS.

### Criteria to declare QTL associated with BCWD resistance

The results from the GWAS performed with BayesB and wssGBLUP were used to identify genomic windows and QTL associated with BCWD resistance. A two stage approach was used to identify a QTL associated with BCWD resistance. First, the genomic windows with explained genetic variance (EGV) greater than 1% and 2% in the TLUM and NCCCWA populations, respectively, were declared as genomic regions associated with BCWD resistance. The threshold to declare a QTL was raised to EGV ≥ 2% in the relatively small NCCCWA sample (*n* = 577) to control the type I error rate. Second, to determine if neighboring or overlapping windows on the same chromosome belong to the same QTL region we used the following criteria: all windows associated with BCWD resistance that were bounded within a region smaller than 20 Mb and were less than 10 Mb apart from another associated window were grouped to a single QTL region.

The BayesB algorithm estimated the proportion of models where the tested 1 Mb-window was included as with non-zero variance (*p* > 0) and therefore accounted for more than 0% of the genetic variance. These *p* > 0 estimates were used to calculate the probability of false positives [*PFP* = 1 − (*p* > 0)] for each tested window (Fernando and Garrick, 2013). These PFP estimates enabled accounting for multiple testing to control the probability of false positive conclusions across all the undertaken GWAS tests with the BayesB model. Thus, by using these EGV thresholds (Peters et al., 2013) and PFP estimates (Fernando and Garrick, 2013), the claims on QTL findings was conservatively restricted to the strongest associations with BCWD resistance to control the type I error rate in this study.

### QTL segregating in both NCCCWA and TLUM populations

In order to identify QTL that might be overlapping between the two populations and between the two genotyping platforms in the NCCCWA population, we assigned the genome physical map positions to all flanking markers of the genomic windows associated with BCWD resistance using the rainbow trout reference genome sequence (GenBank Assembly Accession GCA_002163495) and searched for overlapping QTL regions within each chromosome using the flanking markers physical map genome coordinates.

## Results

### Heritability of BCWD resistance

The BCWD mortality rate were 0.55 and 0.70 in TLUM and NCCCWA populations, respectively. The heritability or proportion of phenotypic variance explained by the markers for survival DAYS and the binary survival STATUS were previously reported (Vallejo et al., 2016, 2017). Briefly, the heritability were moderate with a range of 0.24–0.34 and 0.23–0.35 for DAYS and STATUS, respectively (Table 1); and the mean heritability for DAYS and STATUS were similar (0.29). The heritability of STATUS estimated on the underlying scale of liability using a threshold model was transformed to the observed scale of disease survival STATUS using already described procedures (Vallejo et al., 2017). Overall, for both BCWD phenotypes and across genotyping platforms and populations, the mean heritability estimated with wssGBLUP (0.32) was slightly higher than that estimated with BayesB (0.27).

### QTL associated with BCWD resistance in the TLUM population (57 K SNP chip)

We have previously shown that the BCWD resistance phenotypes DAYS and STATUS yielded similar results in QTL mapping (Vallejo et al., 2014a; Liu et al., 2015b; Palti et al., 2015b) and genomic selection experiments (Vallejo et al., 2016, 2017). In this study, we observed also that STATUS and DAYS were affected by similar QTL regions with few exceptions (Overall, DAYS detected three more QTL than STATUS) (Table S2). However, because STATUS resembles the disease resistance trait closer than DAYS; and selection programs for improved disease resistance would most likely favor improvement of resistance over endurance or tolerance (Ødegård et al., 2011), we present the results from the STATUS survival phenotype in the main body of this report. The complete results from the analysis with the DAYS phenotype are presented in the Supplementary Material section.

**Figure 1:**
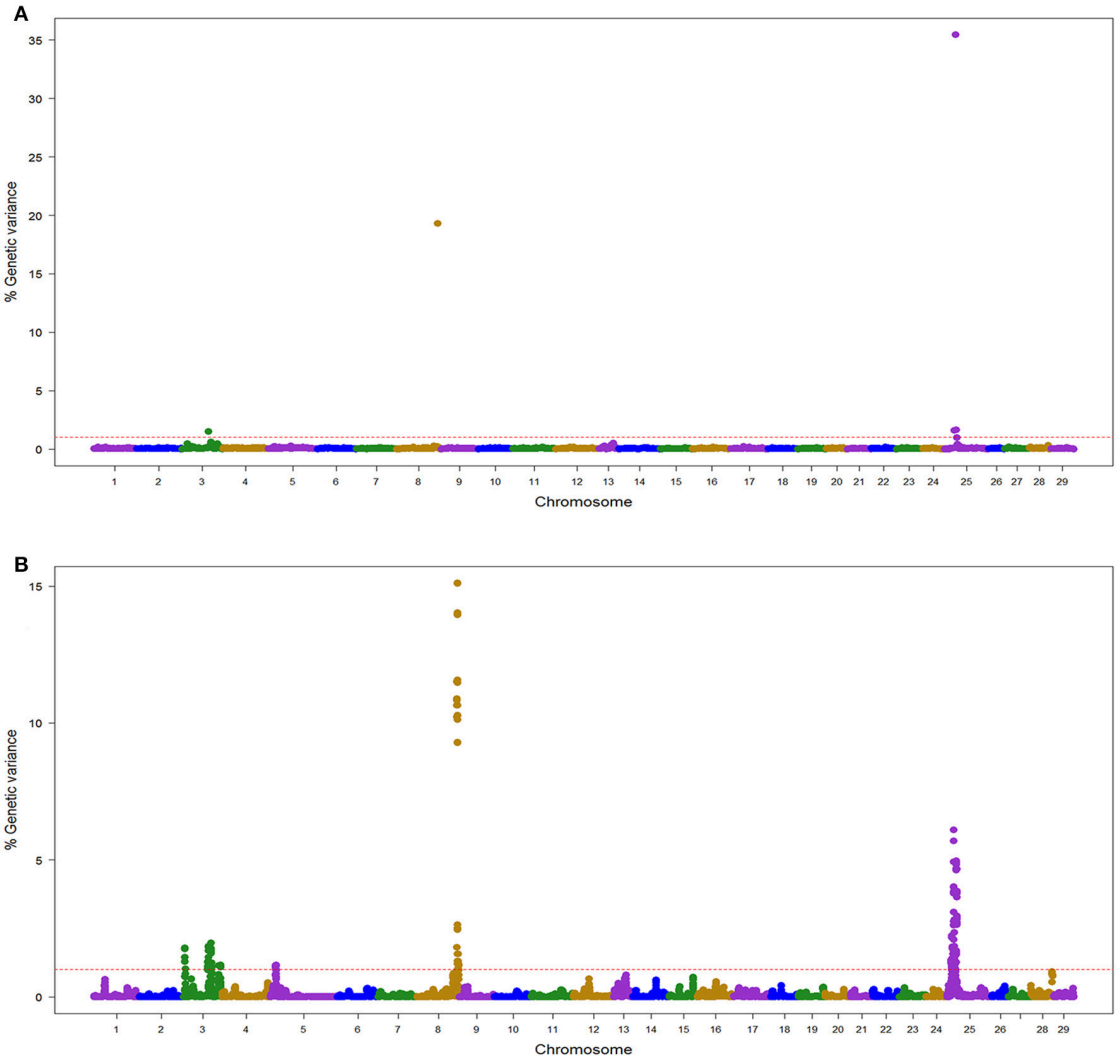
Manhattan plot showing the association between SNP genomic windows and BCWD resistance in TLUM sample genotyped with 57 K Chip-SNP: **(A)** GWAS for STATUS performed with BayesB using 1 Mb exclusive windows. **(B)** GWAS for STATUS performed with wssGBLUP using 1 Mb sliding windows.

In the TLUM population, a total of 45 windows with EGV ≥1% were detected on chromosomes Omy3, 5, 8, 13, and 25 (Table 2; Figure 1; Table S2 and Figure S1). Fourteen windows were detected with BayesB with EGV up to 57.6% and 31 windows were detected with wssGBLUP with EGV up to 28.7%. Four QTL (3.2, 8.1, 13.2, and 25.1) were detected by both GWAS models (Table S2). We did not detect any BayesB model specific QTL (i.e., all QTL detected with BayesB were also detected with wssGBLUP); and four QTL (3.1, 3.3, 5.1, and 13.1) were detected only with wssGBLUP. Overall, we detected eight QTL (3.1, 3.2, 3.3, 5.1, 8.1, 13.1, 13.2, and 25.1) associated with BCWD resistance which jointly explained up to 61% of the genetic variance for BCWD resistance in the TLUM population when accounting only for the largest EGV window in each QTL (Table 2; Tables S2, S3). Among these eight QTL, two significant large-effect QTL were detected on Omy8 (QTL 8.1; *PFP* = 0.0) and 25 (QTL 25.1; *PFP* = 0.01) with BayesB, each explaining up to 19.3 and 35.4% of the genetic variance for BCWD resistance, respectively (Table 2).

**Table 2:** Summary of QTL associated with BCWD survival STATUS in the Troutlodge US May (TLUM) population^a^.

**Omy**	**QTL^b^**	**GWAS method^c^**	**Genetic variance (%)^d^**	**Physical Map (bp)^e^**		**Markers in Window**		**SNPs per window**
				**Start**	**End**	**Start-SNP**	**End-SNP**	
3	3.1	wssGBLUP	1.8	17,812,341	18,600,963	Affx-88909970	Affx-88904917	22
3	3.2	wssGBLUP	2.0	61,621,949	62,558,467	Affx-88925949	Affx-88919479	18
3	3.3	wssGBLUP	1.2	77,108,538	78,076,592	Affx-88925305	Affx-88929879	19
5	5.1	wssGBLUP	1.2	11,339,155	12,329,117	Affx-88916119	Affx-88936955	27
8	8.1	BayesB	19.3	76,070,399	76,907,400	Affx-88955037	Affx-88906927	17
25	25.1	BayesB	35.4	21,006,787	21,805,909	Affx-88924154	Affx-88936445	30

a *The fish from TLUM population were genotyped with the 57 K SNP array (Chip)*.

b *From each QTL, the window with the highest explained genetic variance is presented in this Table. The QTL nomenclature was based on chromosome number and physical genome map positions of the SNPs that flanked each QTL region within the chromosome, where the region with the lowest position numbers determined to be QTL1, the next QTL2 and so on (i.e., Omy3: QTL 3.1, 3.2, etc.)*.

c *GWAS was conducted using Bayesian variable selection model BayesB (BayesB) and weighted single-step GBLUP (wssGBLUP) methods. BayesB used 1 Mb exclusive-consecutive windows and wssGBLUP used 1 Mb moving-sliding windows*.

d *Explained genetic variance by tested window (%)*.

e *SNP positions in base pairs (bp) based on rainbow trout reference genome sequence (GenBank Assembly Accession GCA_002163495)*.

### QTL for BCWD resistance in the NCCCWA population (57 K SNP chip)

**Figure 2:**
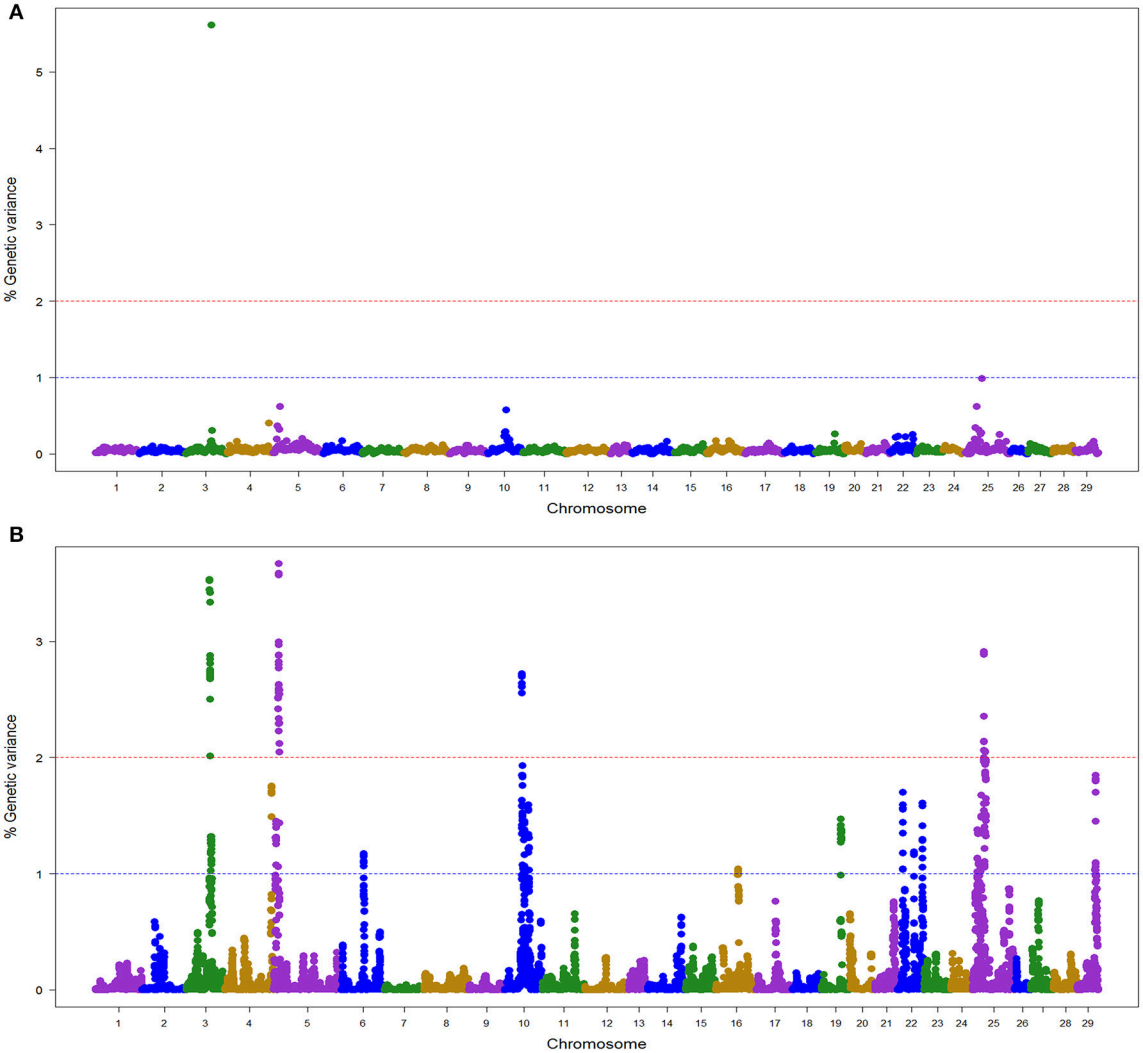
Manhattan plot showing the association between SNP genomic windows and BCWD resistance in NCCCWA sample genotyped with 57 K Chip-SNP: **(A)** GWAS for STATUS performed with BayesB using 1 Mb exclusive windows. **(B)** GWAS for STATUS performed with wssGBLUP using 1 Mb sliding windows.

In the NCCWA population using the 57 K SNP chip, we detected a total of 11 windows associated with BCWD resistance on chromosomes Omy3, 5, 10, 22, and 25 (Table 3; Figure 2; Table S2 and Figure S2). Two and nine QTL windows were detected with BayesB and wssGBLUP, respectively, and each GWAS model explained up to 5.6 and 16.1% of the genetic variance, respectively.

**Table 3:** Summary of QTL associated with BCWD survival STATUS in NCCCWA population detected using the 57 K SNP array.

**Omy**	**QTL^a^**	**GWAS method^b^**	**Genetic variance (%)^c^**	**Physical Map (bp)^d^**		**Markers in Window**		**SNPs per window**
				**Start**	**End**	**Start-SNP**	**End-SNP**	
3	3.2	BayesB	5.6	55,025,670	55,964,831	Affx-88917670	Affx-88935875	24
5	5.1	wssGBLUP	3.7	11,245,430	12,244,569	Affx-88930371	Affx-88921454	36
10	10.1	wssGBLUP	2.7	31,536,788	32,517,865	Affx-88925834	Affx-88904643	47
25	25.1	wssGBLUP	2.9	28,240,466	29,219,522	Affx-88919589	Affx-88945013	40

a *From each QTL, the window with the highest explained genetic variance is presented in this Table. The QTL nomenclature was based on chromosome number and physical genome map positions of the SNPs that flanked each QTL region within the chromosome, where the region with the lowest position numbers determined to be QTL1, the next QTL2 and so on (i.e., Omy3: QTL 3.1, 3.2, etc.)*.

b *GWAS was conducted using Bayesian variable selection model BayesB (BayesB) and weighted single-step GBLUP (wssGBLUP) methods. BayesB used 1 Mb exclusive-consecutive windows and wssGBLUP used 1 Mb moving-sliding windows*.

c *Explained genetic variance by tested window (%)*.

d *SNP positions in base pairs (bp) based on rainbow trout reference genome sequence (GenBank Assembly Accession GCA_002163495)*.

Only one QTL (3.2) was detected by both GWAS models (Table S2). We did not detect any BayesB model specific QTL, and four QTL (5.1, 10.1, 22.1, and 25.1) were detected only with the wssGBLUP method.

Overall, we detected five QTL (3.2, 5.1, 10.1, 22.1, and 25.1) associated with BCWD resistance that explained up to 18.2% of the genetic variance in the NCCCWA population when accounting only for the largest EGV window in each QTL (Table 3 and Table S4).

### QTL for BCWD resistance in the NCCCWA population detected with RAD SNPs

**Figure 3:**
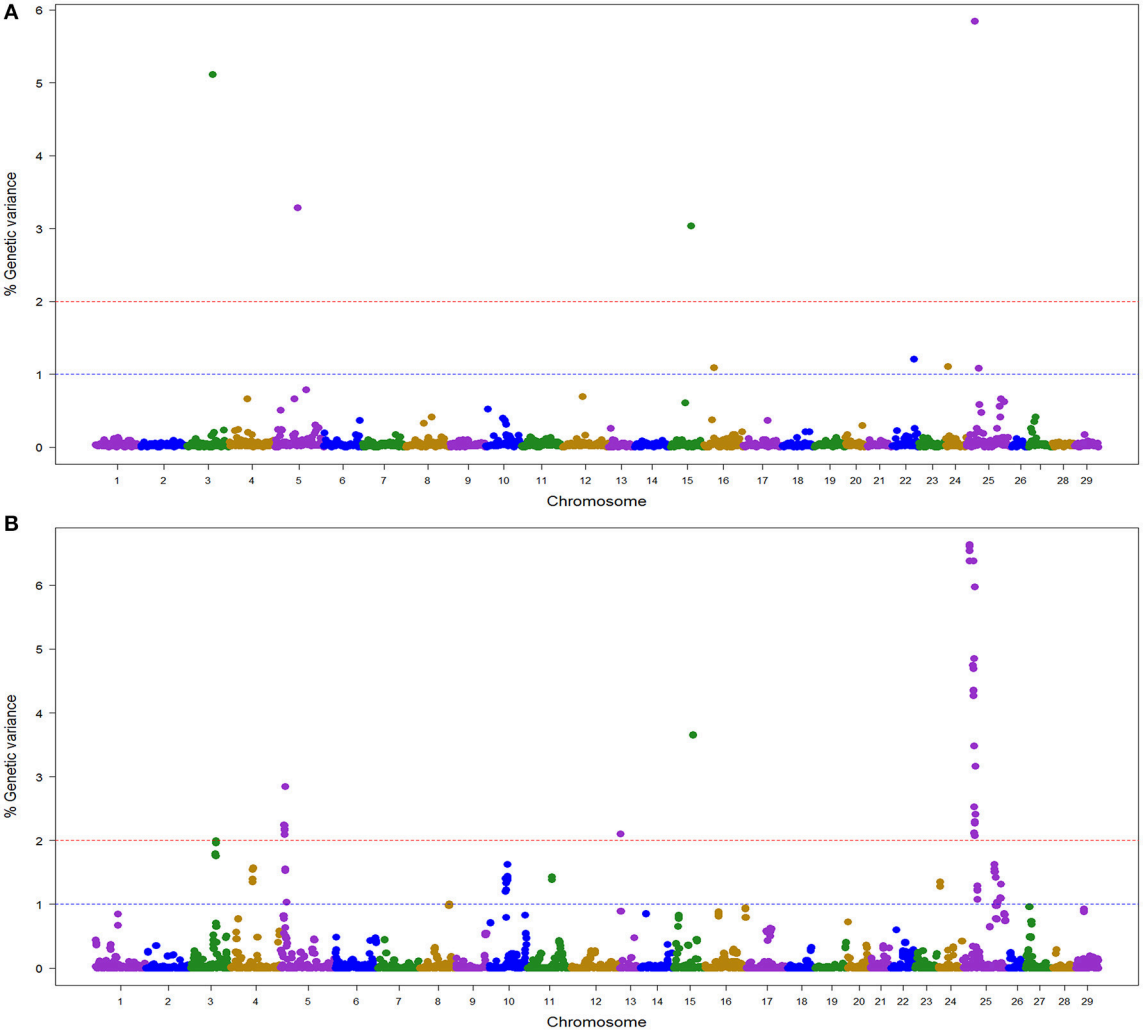
Manhattan plot showing the association between SNP genomic windows and BCWD resistance in NCCCWA sample genotyped with the RAD-SNPs: **(A)** GWAS for STATUS performed with BayesB using 1 Mb exclusive windows. **(B)** GWAS for STATUS performed with wssGBLUP using 1 Mb sliding windows.

In the NCCCWA population using the RAD SNP genotypes, we detected a total of 18 windows associated with BCWD resistance on chromosomes Omy3, 5, 10, 11, 13, 15, and 25 (Table 4; Figure 3; Table S5 and Figure S3). Four windows were detected by BayesB with EGV up to 17.3% and 14 windows were detected by wssGBLUP with EGV up to 26.4%.

**Table 4:** Summary of QTL associated with BCWD survival STATUS in NCCCWA population detected using RAD-SNPs genotyping.

**Omy**	**QTL^a^**	**GWAS method^b^**	**Genetic variance (%)^c^**	**Physical Map (bp)^d^**		**Markers in Window**		**SNPs per window**
				**Start**	**End**	**Start-SNP**	**End-SNP**	
3	3.2	BayesB	5.1	55,254,048	55,993,431	BCWD10F04977	BCWD10F00765	3
5	5.1	wssGBLUP	2.8	11,966,155	12,948,479	BCWD10F15578	BCWD10F00357	11
5	5.2	BayesB	3.3	41,094,726	41,686,801	BCWD10F02753	BCWD10F18861	6
13	13.1	wssGBLUP	2.1	11,230,016	11,602,309	BCWD10F05067	BCWD10F24318	3
15	15.1	wssGBLUP	3.7	38,446,758	39,349,228	BCWD10F00773	BCWD10F16617	3
25	25.1	wssGBLUP	6.6	17,496,495	18,444,865	BCWD10F06483	BCWD10F14339	11

a *From each QTL, the window with the highest explained genetic variance is presented in this Table. The QTL nomenclature was based on chromosome number and physical genome map positions of the SNPs that flanked each QTL region within the chromosome, where the region with the lowest position numbers determined to be QTL1, the next QTL2 and so on (i.e., Omy3: QTL 3.1, 3.2, etc.)*.

b *GWAS was conducted using Bayesian variable selection model BayesB (BayesB) and weighted single-step GBLUP (wssGBLUP) methods. BayesB used 1 Mb exclusive-consecutive windows and wssGBLUP used 1 Mb moving-sliding windows*.

c *Explained genetic variance by tested window (%)*.

d *SNP positions in base pairs (bp) based on rainbow trout reference genome sequence (GenBank Assembly Accession GCA_002163495)*.

Three QTL (3.2, 15.1, and 25.1) were detected by both GWAS models (Table S2). The QTL 5.2 was detected only with BayesB, and five QTL (5.1, 10.1, 11.1, 13.1, and 25.2) were detected only with wssGBLUP.

Overall, we detected nine QTL (3.2, 5.1, 5.2, 10.1, 11.1, 13.1, 15.1, 25.1, and 25.2) associated with BCWD resistance which explained up to 31.9% of the genetic variance in this NCCCWA population dataset when accounting only for the largest EGV window in each QTL (Table 4 and Table S5).

## Discussion

In GWAS studies, the use of correct statistical models and computer algorithms is paramount to successfully identify the underlying genetic basis of resistance to complex diseases in livestock and aquaculture species. To date there have been several reported GWAS using single-marker association tests in fin fish species (Campbell et al., 2014; Ayllon et al., 2015; Geng et al., 2015; Gonen et al., 2015; Liu et al., 2015b; Palti et al., 2015b; Tsai et al., 2015, 2016) which generally are associated with high type I error rate because single-marker methods do not account for linkage disequilibrium (LD) between physically linked loci in the association test. In this study, we performed GWAS for loci associated with BCWD resistance using multiple-regression models which estimate the effect of all markers simultaneously and consequently do account for LD between neighboring SNPs (Fernando and Garrick, 2013; Garrick and Fernando, 2013; Misztal et al., 2014).

In the current GWAS, we detected 14 QTL associated with BCWD resistance in two commercially-relevant rainbow trout breeding populations from which 11 were validated QTL from previous studies and three were novel QTL. Here we confirmed that BCWD resistance is controlled by the oligogenic inheritance of few moderate-large effect loci and a large-unknown number of loci with small effects on BCWD resistance. However, despite the similar genetic architecture for this trait in both populations and the detection of overlapping QTL, we still detected major QTL differences between the two populations. We also found that the RAD and Chip genotyping platforms did not detect the same QTL in the NCCCWA population, and overall the RAD platform detected a greater number of QTL than the Chip platform. In addition, the wssGBLUP and the BayesB multiple-regression GWAS models did not detect the same QTL, which highlights the utility of using different GWAS models to effectively optimize the discovery of QTL.

### The genetic architecture of BCWD resistance in rainbow trout

Previously, we predicted that 6–10 QTL explaining 83–89% of phenotypic variance with either additive or dominant disease-resistant alleles plus polygenic effects may underlie the genetic architecture of BCWD resistance in the same NCCCWA population using Bayesian complex segregation analysis of phenotype and pedigree records (Vallejo et al., 2010). In the current study we were able to confirm our prediction on the genetic architecture of the trait. With 10 families from that original NCCCWA population, we uncovered 10 moderate-large effect QTL that explained up to 27.7% of the additive genetic variance for BCWD resistance (Table S2). Similarly, in the TLUM odd-year population, we detected 8 moderate-large effect QTL that explained up to 60.8% of the additive genetic variance for BCWD resistance.

Four QTL regions located on chromosomes Omy3, 5, 13, and 25 are segregating in both populations (Table S6). The shared QTL regions explain a substantial proportion of the additive genetic variance for BCWD resistance in the two populations (up to 18 and 38.6% of the genetic variance in NCCCWA and TLUM, respectively); suggesting a common underlying genetic architecture for BCWD resistance in the two populations. However, major differences were also detected between the two populations. Six QTL, which explained up to 9.7% of the genetic variance and are located on chromosomes Omy5, 10, 11, 15, 22, and 25 were found only in the NCCCWA population (Table S7). Conversely, four QTL which explained up to 22.2% of the genetic variance and are located on chromosomes Omy3, 8, and 13 were only found in the TLUM population. Overall, our GWAS results confirmed the hypothesis that BCWD resistance is controlled by the oligogenic inheritance of several moderate-large effect QTL and many small effect polygenic loci (Vallejo et al., 2010, 2014a; Liu et al., 2015b; Palti et al., 2015b).

Further fine-mapping of the BCWD-QTL position and eventual identification of putative candidate genes or disease-causal mutations would be advantageous for applying marker-based selection and advancing the understanding of the mechanisms of genetic resistance to BCWD in rainbow trout populations. This can be achieved by genotyping and disease testing a greater number of SNPs from positions within and near the major QTL regions, by re-sequencing highly characterized BCWD resistant and susceptible individuals as was successfully done in the search for the IPNV resistance gene in Atlantic salmon (Moen et al., 2015). In addition, positional and functional candidate genes for the QTL can be generated by interrogating the newest version of the rainbow trout reference genome sequence (GenBank Assembly Accession GCA_002163495).

### Comparing the two SNP genotyping technologies in the NCCCWA population

Overall, the RAD genotyping technology (18 windows; EGV = 32.8%; Table S2) detected a greater number of windows associated with BCWD resistance than the Chip technology (11 windows; EGV = 18.2%) in the NCCCWA population. From the 10 QTL found in the NCCCWA population, more than half of the detected QTL were genotyping platform specific: One QTL was detected only with the Chip technology (QTL 22.1); and five QTL were detected only with the RAD technology (QTL 5.2, 11.1, 13.1, 15.1, and 25.2). Four QTL were detected by both SNP genotyping technologies (QTL 3.2, 5.1, 10.1, and 25.1). The overall better performance of RAD-SNPs than Chip-SNPs in detecting QTL associated with BCWD resistance in the NCCCWA population may be due to sample ascertainment bias effects considering that the 57 K SNP Chip was developed using a collection of samples from different rainbow trout populations to maximize SNP polymorphism and discovery (Palti et al., 2015a). Therefore, the polymorphic markers in the SNP Chip might be less informative (i.e., monomorphic chip SNP in the NCCCWA population or not in LD with QTL) than the SNPs we genotyped with the RAD technology, which were specifically discovered in the sampled families from the NCCCWA population, and are therefore more informative for characterizing genome loci polymorphisms in this dataset.

### Comparing BayesB and wssGBLUP models

Overall, we noticed that the wssGBLUP detected higher number of windows (54) associated with BCWD resistance than the BayesB (20) across the three datasets (TLUM-SNP, NCCCWA-SNP and NCCCWA-RAD) we used here (Table S2): BayesB detected 14, 2, and 4 QTL windows and wssGBLUP detected 31, 9, and 14 QTL windows in TLUM-SNP, NCCCWA-SNP, and NCCCWA-RAD datasets, respectively. These results suggests that wssGBLUP is more liberal than BayesB in declaring a QTL so those QTL found with wssGBLUP might have a higher type I error rate than those detected with BayesB. Performing GS with the Chip genotyped SNPs, we have shown that BayesB predicts GEBVs with higher accuracy than wssGBLUP when using a training sample size of *n* = 1,473 (Vallejo et al., 2017); however, wssGBLUP outperforms BayesB when using a smaller training sample size of *n* = 583 (Vallejo et al., 2016). So, in agreement with these previous GS results, it seems also that the QTL detection power of GWAS with BayesB is more sensitive to sample size reduction than wssGBLUP. We think that the difference in power robustness to sample size reduction of these GWAS models is due to their algorithmic differences. The BayesB model does not explicitly use the available pedigree information and includes in the analysis only animals that had both genotype and phenotype records and as a consequence is less power robust to sample size reduction and needs a minimum sample size for optimal performance, i.e., *n* ≥ 1,000 animals (Garrick and Fernando, 2013). In contrast, the power of GWAS analysis with wssGBLUP is more robust to sample size reduction because it uses the available pedigree information and includes in the analysis animals that have both phenotype and genotype records, and also animals that have only phenotype records (those with missing genotype data).

In the Chip-SNP genotyped TLUM sample, four QTL (3.2, 8.1, 13.2, and 25.1) were detected with both GS models, and four QTL (3.1, 3.3, 5.1, and 13.1) were detected only with wssGBLUP (Table S2). Similarly, in the Chip-SNP genotyped NCCCWA sample, only the QTL 3.2 was detected with both GS models, and most of the QTL (5.1, 10.1, 22.1, and 25.1) were detected only with wssGBLUP. In contrast, in the RAD-SNP genotyped NCCWA sample, three QTL (3.2, 15.1, and 25.1) were detected with both GS models, the QTL 5.2 was detected only with BayesB, and five QTL (5.1, 10.1, 11.1, 13.1, and 25.2) were detected only with wssGBLUP. Thus, these results highlight the importance of using at least two different GWAS algorithms to efficiently uncover the underlying genetic basis of resistance against BCWD in the studied populations.

### Comparing QTL detected in TLUM and NCCCWA populations

In spite of the smaller sample size of the NCCCWA population in comparison to the TLUM population, we detected 10 QTL in the NCCCWA population and only eight QTL in the TLUM population (Table S2). We hypothesize that because the TLUM sample size was much larger than the NCCCWA sample size, it is likely that the type I error rate was smaller in the TLUM sample than in the NCCCWA sample. Therefore, we predict that most of the QTL detected in the TLUM population are real, but some of the QTL detected in the NCCCWA population are false positives. Specifically, the NCCCWA population had two unique QTL (5.2 and 15.1) that were also not reported in past studies. Thus, those two NCCCWA-specific QTL may very well be false positives.

### Comparing our GWAS results with previous studies

Eleven of the 14 QTL we detected in this study were also reported in previous studies in the NCCCWA germplasm and in other populations (Table S8) (Johnson et al., 2008; Wiens et al., 2013; Campbell et al., 2014; Quillet et al., 2014; Vallejo et al., 2014a,b; Liu et al., 2015b; Palti et al., 2015b). Campbell et al. (2014) detected RAD SNPs associated with BCWD resistance in another commercial rainbow trout population that were about 1 Mb from our QTL Omy8.1 (Tables S2, S8); they also reported QTL that overlap or are close to our detected QTL 10.1 and 25.2. Kutyrev et al. (2016) measured the expression of immune relevant genes on spleen tissue sampled from BCWD resistant (ARS-Fp-R) and susceptible (ARS-Fp-S) genetic lines after laboratory disease pathogen challenge and detected differential expression between the tested genetic lines for genes *il1r-like-1* and *tnfrsf1a-like-a*. Interestingly, two SNPs for the gene *il1r-like-1* (Affx-88933101 and Affx-88915186) were about 240 Kb from our QTL 3.2 which explained up to 5.6% of the genetic variance in the NCCCWA population.

Previous studies also detected QTL for BCWD resistance on chromosomes Omy1 (Vallejo et al., 2014a; Palti et al., 2015b), 2 (Vallejo et al., 2014a; Liu et al., 2015b), 7 (Quillet et al., 2014; Vallejo et al., 2014a; Palti et al., 2015b), 12 (Vallejo et al., 2014a; Liu et al., 2015b), 17 (Johnson et al., 2008; Campbell et al., 2014; Quillet et al., 2014), 26, and 28 (Liu et al., 2015b) which were not detected in this study. These conflicting results in QTL mapping can be expected due to several reasons including: (1) QTL effects can be population and/or family specific with unique extent/phase of linkage between QTL and marker alleles; and (2) they can also represent false positive results due to limitations and weaknesses of experimental-design and power of analysis as we describe here.

## Conclusion

This GWAS is the most comprehensive genome-wide scan for QTL associated with BCWD resistance performed to date in two commercially-relevant rainbow trout breeding populations, using two whole-genome SNP genotyping platforms and two multiple-regression GWAS models. We identified a total of 14 moderate-large effect QTL associated with resistance to BCWD resistance, and four of those QTL were segregating in the two populations. These GWAS results confirmed that the genetic architecture of BCWD resistance is controlled by the oligogenic inheritance of few moderate-large effect genes and many small effect resistance loci. Overall, the wssGBLUP detected higher number of QTL than the BayesB and both GWAS models did not detect the same QTL which highlights the utility of using two different GWAS algorithms to effectively discover QTL. The RAD genotyping platform detected higher number of QTL than the Chip technology and also both genotyping platforms did not detect the same QTL in the NCCCWA population. These GWAS results will advance the biological and functional analysis of positional candidate genes using the annotation of the new rainbow trout reference genome (GenBank Assembly Accession GCA_002163495). They will also be used for evaluating and implementing more efficient selective breeding strategies which will utilize the QTL-flanking SNPs in genome-enabled selection for BCWD resistance in rainbow trout aquaculture.

## Author contributions

YP, TL and RV conceived and planned the study; YP, GW, JE, TL and TW coordinated, supervised and performed the disease challenges and samples collection; YP coordinated and supervised samples processing, RAD libraries preparation and RAD and Chip genotyping; AH performed RAD libraries sequencing; GG performed genotype data quality control and bioinformatics filtering and developed a database pipeline to assemble genotype and phenotype records; SL performed RAD genotyping data analysis; KM and JP contributed tissue samples and live fish from the Troutlodge, Inc. May nucleus breeding population; TL, JP and KM provided pedigree records and identified the fish for genotyping based on pedigree and phenotype records; RV executed the statistical data genetic and GWAS analyses and wrote the first draft of this document; and BF provided support in performing weighted single-step GWAS analysis. All authors read and approved the final manuscript.

### Conflict of interest statement

JP and KM were employed by the company Troutlodge, Inc. The other authors declare that the research was conducted in the absence of any commercial or financial relationships that could be construed as a potential conflict of interest. The reviewer DN declared a shared affiliation, though no other collaboration, with the authors RV, GG, TL, JE, TW, GW and YP to the handling Editor.

## Supplementary material

Figure S1 Manhattan plot showing the association between SNP genomic windows and BCWD resistance in TLUM sample genotyped with 57 K Chip-SNP: **(A)** GWAS for DAYS performed with BayesB using 1 Mb exclusive windows. **(B)** GWAS for DAYS performed with wssGBLUP using 1 Mb sliding windows.

Figure S2 Manhattan plot showing the association between SNP genomic windows and BCWD resistance in NCCCWA sample genotyped with 57 K Chip-SNP: **(A)** GWAS for DAYS performed with BayesB using 1 Mb exclusive windows. **(B)** GWAS for DAYS performed with wssGBLUP using 1 Mb sliding windows.

Figure S3 Manhattan plot showing the association between SNP genomic windows and BCWD resistance in NCCCWA sample genotyped with the RAD-SNPs: **(A)** GWAS for DAYS performed with BayesB using 1 Mb exclusive windows. **(B)** GWAS for DAYS performed with wssGBLUP using 1 Mb sliding windows.
