# Supplementary figures and images for: Similar Genetic Architecture with Shared and Unique Quantitative Trait Loci for Bacterial Cold Water Disease Resistance in Two Rainbow Trout Breeding Populations
Source: Front Genet. 2017 Oct 23;8:156. doi: 10.3389/fgene.2017.00156 (PMC5660510; doi:10.3389/fgene.2017.00156)

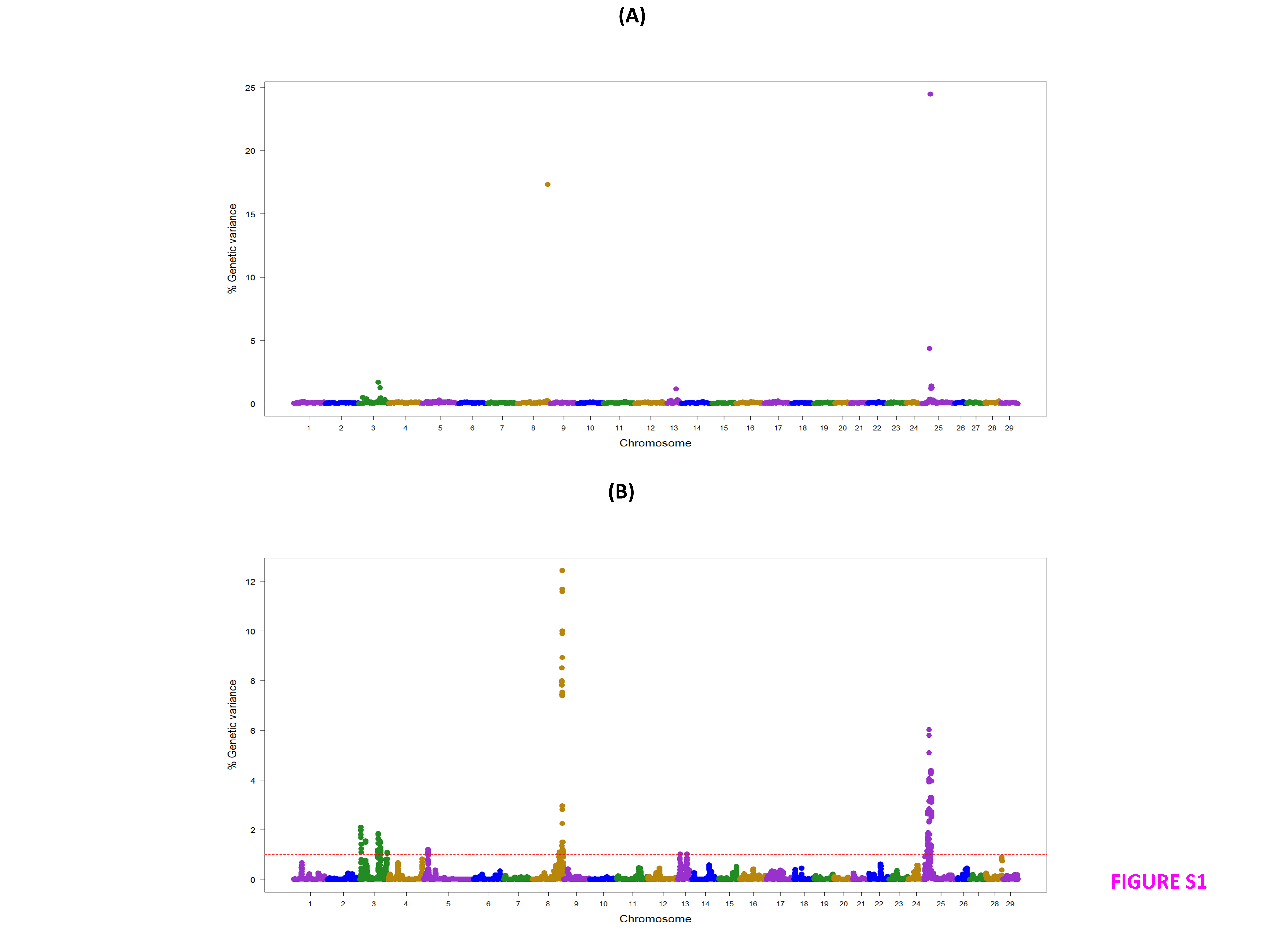

Supplement: Figure S1 — Manhattan plot showing the association between SNP genomic windows and BCWD resistance in TLUM sample genotyped with 57 K Chip-SNP: (A) GWAS for DAYS performed with BayesB using 1 Mb exclusive windows. (B) GWAS for DAYS performed with wssGBLUP using 1 Mb sliding windows. [file Image1.TIF]

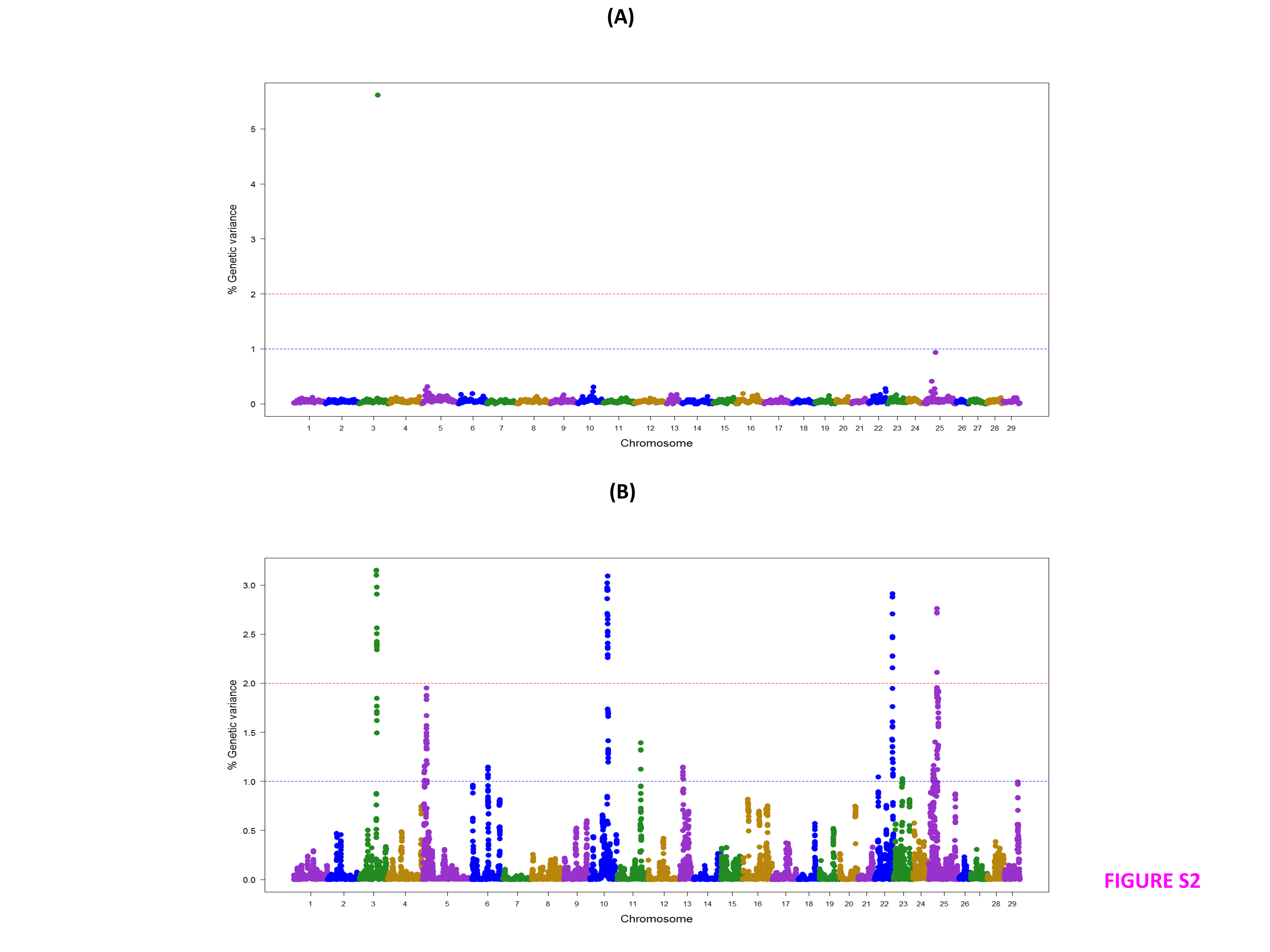

Supplement: Figure S2 — Manhattan plot showing the association between SNP genomic windows and BCWD resistance in NCCCWA sample genotyped with 57 K Chip-SNP: (A) GWAS for DAYS performed with BayesB using 1 Mb exclusive windows. (B) GWAS for DAYS performed with wssGBLUP using 1 Mb sliding windows. [file Image2.TIF]

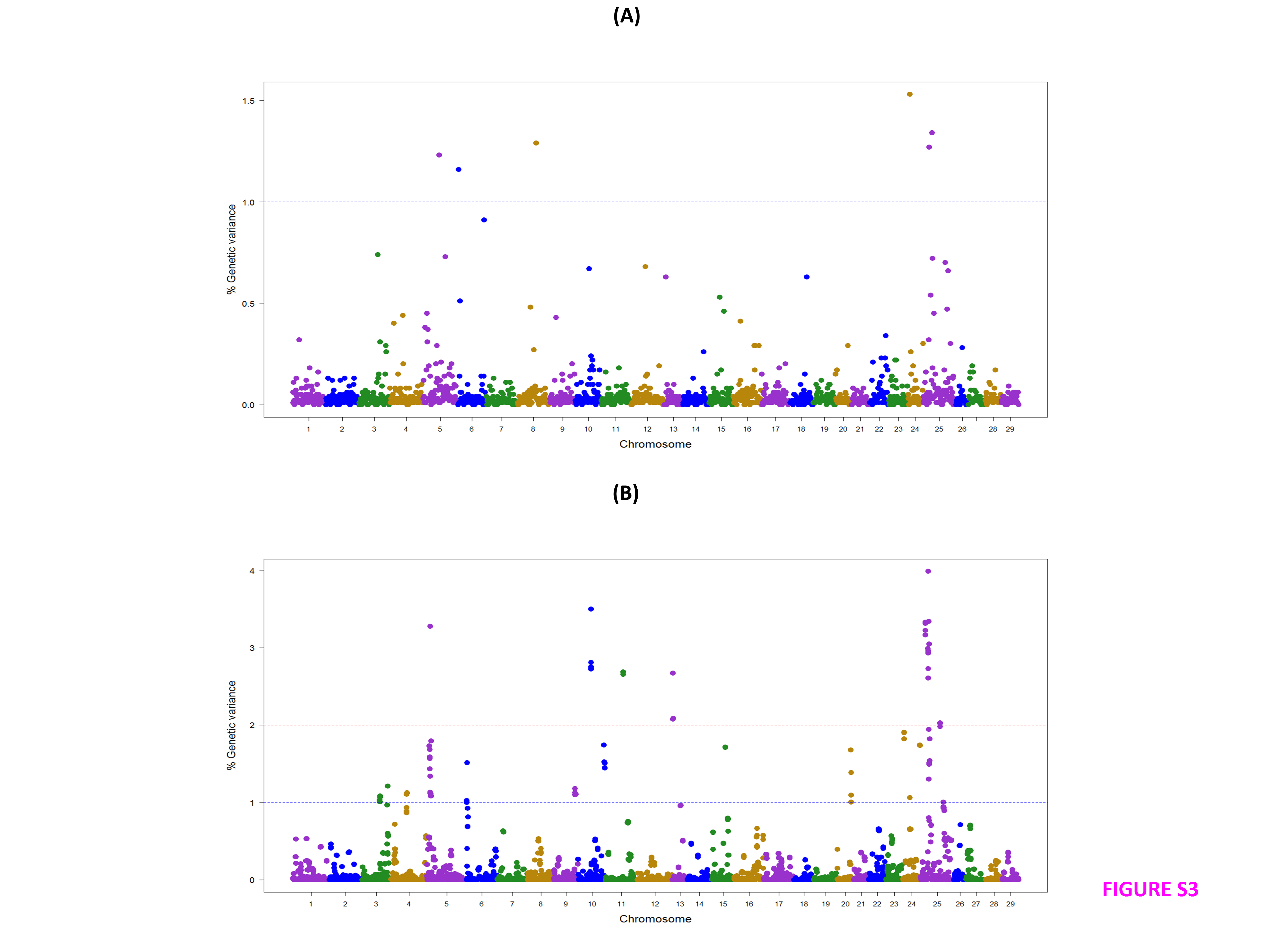

Supplement: Figure S3 — Manhattan plot showing the association between SNP genomic windows and BCWD resistance in NCCCWA sample genotyped with the RAD-SNPs: (A) GWAS for DAYS performed with BayesB using 1 Mb exclusive windows. (B) GWAS for DAYS performed with wssGBLUP using 1 Mb sliding windows. [file Image3.TIF]
