# Supplementary material for: Similar Genetic Architecture with Shared and Unique Quantitative Trait Loci for Bacterial Cold Water Disease Resistance in Two Rainbow Trout Breeding Populations
Source: Front Genet. 2017 Oct 23;8:156. doi: 10.3389/fgene.2017.00156 (PMC5660510; doi:10.3389/fgene.2017.00156)
Supplement: Supplementary file 8 [file Table5.docx]

**ADDITIONAL TABLE S5 |** Summary of QTL associated with BCWD survival DAYS in NCCCWA population detected using RAD-SNPs genotyping.

| Omy | QTL*^a^* | GWAS method*^b^* | Genetic variance (%)*^c^* | Physical Map (bp)*^d^* | |  | Markers in Window | | SNPs per window |
| --- | --- | --- | --- | --- | --- | --- | --- | --- | --- |
|  |  |  |  | **Start** | **End** |  | **Start-SNP** | **End-SNP** |  |
| 5 | 5.1 | wssGBLUP | 3.3 | 11,966,155 | 12,948,479 |  | BCWD10F15578 | BCWD10F00357 | 11 |
| 10 | 10.1 | wssGBLUP | 3.5 | 33,038,094 | 33,447,676 |  | BCWD10F12600 | BCWD10F04162 | 5 |
| 11 | 11.1 | wssGBLUP | 2.7 | 47,964,453 | 48,954,610 |  | BCWD10F05737 | BCWD10F22636 | 6 |
| 13 | 13.1 | wssGBLUP | 2.7 | 11,230,016 | 11,602,309 |  | BCWD10F05067 | BCWD10F24318 | 3 |
| 25 | 25.1 | wssGBLUP | 4.0 | 23,817,903 | 24,762,141 |  | BCWD10F02586 | BCWD10F03625 | 9 |
| 25 | 25.2 | wssGBLUP | 2.0 | 56,222,628 | 57,222,099 |  | BCWD10F09466 | BCWD10F22165 | 6 |

*^a^*From each QTL, the window with the highest explained genetic variance is presented in this Table. The QTL nomenclature was based on chromosome number and physical genome map positions of the SNPs that flanked each QTL region within the chromosome, where the region with the lowest position numbers determined to be QTL1, the next QTL2 and so on (i.e., Omy3: QTL 3.1, 3.2, etc.).

*^b^*GWAS was conducted using Bayesian variable selection model BayesB (BayesB) and weighted single-step GBLUP (wssGBLUP) methods. BayesB used 1Mb exclusive-consecutive windows and wssGBLUP used 1Mb moving-sliding windows.

*^c^*Explained genetic variance by tested window (%).

*^d^*SNP positions in base pairs (bp) based on rainbow trout reference genome sequence (GenBank assembly Accession GCA_002163495).
